# Mechanical, Physical, and Chemical Properties of Mycelium-Based Composites Produced from Various Lignocellulosic Residues and Fungal Species

**DOI:** 10.3390/jof8111125

**Published:** 2022-10-25

**Authors:** Worawoot Aiduang, Jaturong Kumla, Sirasit Srinuanpan, Wandee Thamjaree, Saisamorn Lumyong, Nakarin Suwannarach

**Affiliations:** 1 Applied Microbiology (International Program), Department of Biology, Faculty of Science, Chiang Mai University, Chiang Mai 50200, Thailand; 2 Department of Biology, Faculty of Science, Chiang Mai University, Chiang Mai 50200, Thailand; 3 Research Center of Microbial Diversity and Sustainable Utilization, Chiang Mai University, Chiang Mai 50200, Thailand; 4 Department of Physics and Materials Science, Faculty of Science, Chiang Mai University, Chiang Mai 50200, Thailand; 5 Academy of Science, The Royal Society of Thailand, Bangkok 10300, Thailand

**Keywords:** agricultural residues, bio-fabrication, biodegradable materials, fungal mycelium, residues valorization

## Abstract

Mycelium-based composites (MBCs) are characterized as biodegradable materials derived from fungal species. These composites can be employed across a range of industrial applications that involve the manufacturing of packaging materials as well as the manufacturing of buildings, furniture, and various other household items. However, different fungal species and substrates can directly affect the functional properties of MBCs, which ultimately vary their potential to be used in many applications. In this study, the mechanical, physical, and chemical properties of MBCs made from four different fungal species (*Ganoderma fornicatum*, *Ganoderma williamsianum*, *Lentinus sajor-caju*, and *Schizophyllum commune*) combined with three different types of lignocellulosic residues (sawdust, corn husk, and rice straw) were investigated. The results indicate that differences in both the type of lignocellulosic residues and the fungal species could affect the properties of the obtained MBCs. It was found that the MBCs obtained from sawdust had the highest degree of density. Moreover, MBCs obtained from *S. commune* with all three types of lignocellulosic residues exhibited the highest shrinkage value. The greatest degree of water absorption was observed in the MBCs obtained from rice straw, followed by those obtained from corn husk and sawdust. Additionally, the thermal degradation ability of the MBCs was observed to be within a range of 200 to 325 °C, which was in accordance with the thermal degradation ability of each type of lignocellulosic residue. The greatest degrees of compressive, flexural, impact, and tensile strength were observed in the MBCs of *G. williamsianum* and *L. sajor-caju*. The results indicate that the MBCs made from corn husk, combined with each fungal species, exhibited the highest values of flexural, impact, and tensile strength. Subsequently, an analysis of the chemical properties indicated that the pH value, nitrogen content, and organic matter content of the obtained MBCs were within the following ranges: 4.67–6.12, 1.05–1.37%, and 70.40–86.28%, respectively. The highest degree of electrical conductivity was observed in MBCs obtained from rice straw. Most of the physical and mechanical properties of the obtained MBCs were similar to those of polyimide and polystyrene foam. Therefore, these composites could be used to further develop relevant strategies that may allow manufacturers to effectively replace polyimide and polystyrene foams in the future.

## 1. Introduction

Agricultural production has expanded by more than threefold in the last 50 years in response to global population growth and increases in food demand [1]. The increase in agricultural production, processing, and consumption has generated a large quantity of lignocellulosic residue each year [2]. Between 2003 and 2013, Asia has been recognized as the greatest producer of global agricultural residue (47%), followed by America (29%), Europe (16%), Africa (6%), and the Oceania region (2%) [3]. Global agricultural residues are expected to rise to around 2.2 billion tons annually by the year 2025 [4]. Additionally, the manufacture of forestry wood products can also generate a large amount of residue (e.g., bark, chips, slabs, and sawdust) [5]. The effective management of agricultural and wood residues has always been a major concern. Generally, residues are burned and dumped in landfills in developing countries, particularly in Asia because these are some of the most convenient and cost-saving methods of residue management. On the other hand, burning has resulted in a range of air pollution problems that involve increasing emissions of carbon monoxide (CO), carbon dioxide (CO_2_), and particulate matter (PM_10_ and PM_2.5_) [6,7]. Air pollution can potentially cause a range of serious health risks, environmental pollution, and economic problems on both local and regional levels across the globe [2,8,9]. Accordingly, multiple new research studies have focused on reducing and recycling agricultural and wood residues into useful and valuable products using a circular bio-economic approach [10]. Both agricultural and wood residues are defined as lignocellulosic components that are comprised of cellulose, hemicellulose, lignin, and other polysaccharides [11]. These residues can be applied in the development of raw materials that can then be used in the production of other high-value-added products including animal feed, biofuels, enzymes, and value-added fine chemicals [12,13,14]. Remarkably, lignocellulosic residues have also been recognized as a source of nutrients that can facilitate microbial growth [14].

Turning lignocellulosic residues into biomaterials is a fundamental component in the process of establishing a circular bio-economic platform [15]. The development of biomaterials, which is largely based on lignocellulosic residues, has various potential advantages that include their diminished environmental impact and the utilization of renewable resources [16]. Mycelium-based composites (MBCs) are a type of biomaterial that holds great potential for the goal of using agricultural residues in specifically beneficial ways and in the broader embrace of fungal biotechnology [17]. Saprobic fungi can degrade lignocellulosic residues into nutrients through certain bio-fabricated processes wherein their mycelia networks can effectively combine substrate particles together [18]. *Ganoderma*, *Pleurotus*, *Pycnoporus*, and *Trametes* are the most commonly used fungal genera in the production of MBCs [19,20,21]. Interestingly, MBCs have successfully been employed in the development of mycelium-based materials that can be applied in the construction of buildings and in the manufacturing of furniture, packaging, and various other household items [20,21,22,23]. These materials have demonstrated their potential in the mission to replace plastics, synthetic foams, and some wood composites with eco-friendly and biodegradable materials that exhibit the appropriate characteristics of ecologically sound sustainable materials [20,21,22,23,24]. Interestingly, MBCs have several major advantages over classical lignocellulosic composites in that they contain greater amounts of chitin and exhibit higher Young’s modulus and lower elongation capabilities [21,25]. Presently, the Ecovative Company sells packaging and board products that are made from MBCs and that are sold on a commercial basis [19]. Foam-like materials made from MBCs are marketed under the name MycoFlex^TM^ [19]. Furthermore, mycelium-based construction materials derived from MBCs have been developed on a laboratory scale in several cumulative forms, including block materials, particle board, acoustic materials, thermal insulation, cladding materials, surface materials (thin sheets and film), and paste materials [19,26,27,28,29,30,31]. MBCs are generated from various forms of lignocellulosic residues and fungal species that are available in each country [27,32]. However, different species of fungi and different substrate types can directly affect the properties of the final products and the functional aspects of the resulting MBCs [23,24,27,32]. Therefore, this study aimed to produce MBCs from different forms of lignocellulosic residues (including sawdust, corn husk, and rice straw) and fungal species (*Ganoderma fornicatum*, *Ganoderma williamsianum*, *Lentinus sajor-caju*, and *Schizophyllum commune*). Prior to this study, there have been no reports on MBCs produced from these four selected fungal species. Therefore, this is the first report of MBCs produced from these fungi. Subsequently, the mechanical (compression, tensile, flexural, and impact strengths), physical (density, average shrinkage, thermal degradation, and water absorption), and chemical (final pH value, electrical conductivity, organic matter content, and nitrogen content) properties of the obtained MBCs were evaluated. The results of this study can provide valuable information in the production of MBCs and can be employed to enhance relevant strategies for the eco-friendly recycling of agricultural residues as well as to fulfill the long-term goal of replacing plastic and foam in the future.

## 2. Materials and Methods

### 2.1. Sources of Fungal Strains and Culture Conditions

Pure cultures of four fungal species, namely, *Lentinus sajor-caju* CMU-NK0427, *Ganoderma fornicatum* CMU-NK0524, *Ganoderma williamsianum* CMU-NK0540, and *Schizophyllum commune* CMU-S01, were obtained from the culture collection of the Research Center of Microbial Diversity and Sustainable Utilization, Faculty of Science, Chiang Mai University, Thailand. All fungal strains were cultivated on potato dextrose agar (PDA; Conda, Madrid, Spain) and incubated at 30 °C for 7 days.

### 2.2. Sources of Lignocellulosic Residues and Preparation

Three different wood and agricultural residues, including the sawdust of rubber tree, corn husk, and rice straw, were selected and used as substrates in this study. These forms of residues were obtained from a sawmill and agricultural areas located in Chiang Mai Province, Thailand. All selected residues were dried in an oven maintained at 60 °C until they were completely dry. Each substrate was then ground in a wood chipper and sieved. Particles of about 5–20 mm in size were collected and used in this study.

### 2.3. Preparation of Mycelium-Based Composites and Mould Design

#### 2.3.1. Inoculum Preparation of Fungal Mycelium

Mycelial inoculum of each fungal species was prepared using sorghum grains. The sorghum grains were washed and boiled for a period of 20 min. After being allowed to cool, 100 g of the boiled grains were put into glass bottles that were plugged with cotton wool and then autoclaved at 121 °C for 20 min. Afterward, the bottles were allowed to cool down to room temperature over a period of 24 h. Subsequently, mycelial plugs (1 × 1 cm) of each fungal species obtained from colonies grown on PDA were then transferred into bottles (5 plugs per bottle). The inoculated bottles were then incubated at 30 °C in darkness. After two weeks of incubation, the sorghum grains were observed to be completely covered in fungal mycelia, which were then used as the inoculum [33].

#### 2.3.2. Mycelial Growth on Substrates

Each substrate was supplemented with 5% rice bran, 1% calcium carbonate, 2% calcium sulfate, and 0.2% sodium sulfate on a dry mass basis [34]. The mixtures were then adjusted to a final relative humidity value of 60% by adding water. Five hundred grams of mixed substrate was placed in polypropylene bags (3.50 inches wide and 12.5 inches long). The bags were then sealed with cotton-plugged polyvinyl chloride pipe rings, covered with pieces of paper, and autoclaved at 121 °C for 60 min. After being allowed to cool down to room temperature over a period of 24 h, five grams of each mycelial inoculum was inoculated onto the top of the substrate of each bag. The ratio of fungal inoculum to substrate mass was 1:100 (*w*/*w*). The inoculated bags were then incubated at 30 °C in darkness [35]. The fungal mycelia were observed to cover the substrate after 14–21 days of incubation.

#### 2.3.3. Mold Preparation

For the compression test, molds were made from cylindrical plastic boxes with a ratio between the diameter and height of 2:1 (86 mm in diameter and 43 mm in height) according to the method described by Elsacker et al. [36]. The mold for the water absorption test was made from a plastic dish that was 85 mm in diameter and 13 mm in height. Moreover, the molds employed for the tensile, flexural, and impact tests were designed from acrylic clear sheets that were cut into a dumbbell-shaped segment (165 × 19 mm, neck 57 × 13 mm, a rectangular shape (127 × 12.7 × 3.2 mm), and a rectangular shape (63.5 × 12.7 × 12.7 mm), respectively, following the standard method of the American Society for Testing and Materials (ASTM) [26,37].

#### 2.3.4. Mycelium-Based Composite Fabrication, Preparation for Testing, and Moisture Content

**Figure 1:**
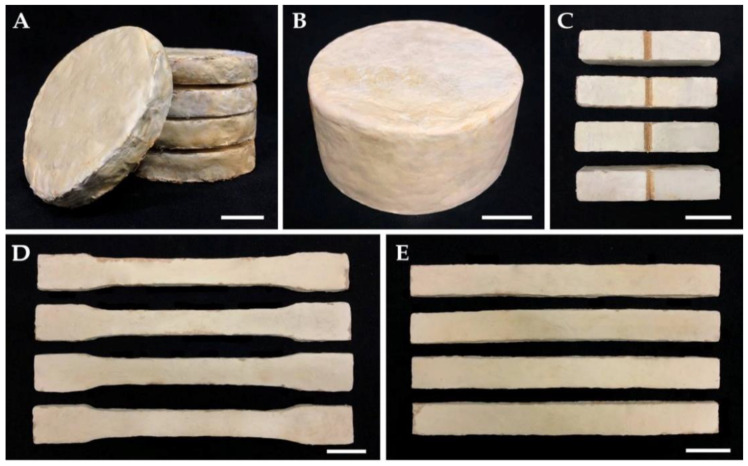
Samples of MBCs obtained from *Lentinus sajor-caju* and corn husk in this study: Samples for water absorption test (**A**). Samples for moisture content, density, shrinkage, and compression tests (**B**). Samples for impact strength test (**C**). Samples for tensile strength test (**D**). Samples for flexural strength test (**E**). Scale bars = 2 cm.

Each substrate that was colonized with each fungal mycelium was put into the mold, pressed using a unidirectional cold press machine (Shop press ZX0901E-1, New Taipei, Taiwan) set at 0.5 MPa for 10 min, and incubated at 30 °C. After three days of incubation, MBCs were removed from the mold and incubated in a plastic box for another three days until the mycelia covered the sides that had come into contact with the mold. The obtained MBCs were then dried in an oven at 70 °C for 24 to 72 h until their mass was stabilized [36]. The moisture content of the MBCs was measured by following the standard method of ASTM D 644 [38]. The percentage moisture content of MBCs was calculated from the percentage of mass loss: Moisture content (%) = [(W1 − W2)/W1] × 100, (where W1 = original mass of the sample and W2 = mass of the sample after being oven dried). Ten replications were performed on each sample for each treatment. The dried MBCs were then kept in desiccators for further experimentation (Figure 1).

### 2.4. Scanning Electron Microscope Observations

All dried MBCs were cut into small squares (5 × 5 mm) using a scalpel. The samples were then attached to a 10 mm^2^ stub adapter with 2 mm^2^ double-sided carbon tape and coated with gold for 2 min under high vacuum mode. Then, the samples were subsequently examined and photographed with a scanning electron microscope (SEM) JEOL JSM-5910 LV SEM (JEOL, Tokyo, Japan) using an accelerating voltage of 15 kV at the Science and Technology Service Center, Faculty of Science, Chiang Mai University, Chiang Mai, Thailand. The surfaces and cross-sectional characteristics of all obtained MBCs were then examined.

### 2.5. Determination of Physical Properties

#### 2.5.1. Density

Density was determined using the MBCs for compression testing. After the MBCs were dried at 70 °C for 24 to 72 h, the density was determined and calculated following the standard method of the International Organization for Standardization (ISO) 9427 [36] by the mass and volume of MBCs. Ten replications were performed on each sample for each treatment.

#### 2.5.2. Water Absorption

The water absorption test was performed according to the standard method of ASTM C272/C272M-18 [39]. Before they were tested, samples of MBCs were dried at 70 °C until their mass was stabilized. The samples were then allowed to cool down in a desiccator for 24 h. The initial mass of the samples was determined. Subsequently, samples were submerged in deionized water for a total duration of 96 h. Samples were then weighed after 12, 24, 36, 48, 60, 72, 84, and 96 h. An increase in mass was calculated by applying the following formula: Mass increasing (%) = [(W − D)/D] × 100, (where W = wet mass, D = dry mass). Each treatment was applied to each sample over ten replications.

#### 2.5.3. Shrinkage

Shrinkage of the samples was determined and calculated based on wet and dry volumes according to the method described by Elsacker et al. [36] and expressed as shrinkage percentage (%) = (V1 − V2/V1) × 100, (where V1 = wet volume of the sample and V2 = dry volume of the sample). Ten replications were completed for each sample in each of the treatments.

#### 2.5.4. Thermal Degradation

The degree of thermal degradation of MBCs was determined by thermogravimetric analysis (TGA) with a thermogravimetric analyzer (Rigaku: Thermo plus EVO2). A mass of each sample of approximately 10 mg was placed in an alumina crucible and heated from 25 to 600 °C at a heating rate of 10 °C/min in a nitrogen atmosphere. Five replications were completed for each sample in each of the treatments.

### 2.6. Determination of Mechanical Properties

#### 2.6.1. Compression Strength

Compression strength was determined following ASTM D 3501 [36] on a Hounsfield-H10Ks (New York, NY, USA) load bench with a 10 kN capacity and a 1 kN load cell under ambient conditions (25 °C with 40 to 50% relative humidity). The tests were conducted with controlled displacement at a rate of 5 mm/min. The load–displacement curve was converted to a stress–strain curve using the following formulas to calculate the compressive stress σ and the strain ε: Stress σ = F/A and Stress ε = ΔL/Lo, respectively (where F: compressive force (N), A: original cross section of the specimen (mm^2^), ΔL: displacement of the loading surfaces (mm), and Lo: original height of the test piece (mm)). Compression strength was reported in MPa units. Ten replications were generated for each of the treatments.

#### 2.6.2. Tensile Strength

Tensile strength was determined following ASTM D 638-14 [26]. The tests were performed with a Hounsfield-H10Ks universal testing machine (New York, NY, USA) using an elongation rate of 2 mm/min and a maximum force of 1 kN. Data were analyzed in order to obtain a stress–strain plot and to provide an indication of tensile strength with ten replications applied to each sample in each treatment.

#### 2.6.3. Flexural Strength

Flexural strength was determined following ASTM D 790-10 [26]. The flexural test was performed with a Hounsfield-H10Ks universal testing machine (New York, NY, USA) employing a three-point bending setup that employed the same machine using a cross-head speed of 2 mm/min and clamp support distance of 40 mm. Ten replications were performed on each sample in each of the treatments.

#### 2.6.4. Impact Strength

Impact strength was determined by employing the Charpy impact test according to the standard of ASTM D-256 [37]. Samples were loaded into the machine and exposed to the pendulum until being fractured. The impact strength values were calculated by dividing the energy by the cross-sectional area of the sample using the following formula: Impact strength (kJ/m^2^) = K/A (where K = energy required to fracture the sample (kJ) and A = cross-sectional area (m^2^)). Ten replications were performed on each sample in each of the treatments.

### 2.7. Determination of Chemical Properties

Samples of MBCs were ground into small pieces in a blender and sieved to less than 2 mm in particle size. Five grams of each sample was soaked in 50 mL distilled water for 30 min. Electrical conductivity (EC) and pH value were measured with the use of conductivity and pH meters, respectively. Moreover, total organic matter and nitrogen content were determined according to the procedure described by Walkley and Black [40] and the method employed by Kjeldahl [41], respectively, at the Agricultural Technology Services Center, Faculty of Agriculture, Chiang Mai University, Ching Mia, Thailand. Five replications were performed for all samples of each treatment.

### 2.8. Statistical Analysis

The data of each experiment were analyzed by one-way analysis of variance (ANOVA) using the SPSS program version 16.0 for Windows. Duncan’s multiple range test was then used to identify any significant differences (*p* ≤ 0.05) between the mean values.

## 3. Results and Discussion

### 3.1. Scanning Electron Microscope Observations and Moisture Content

**Figure 2:**
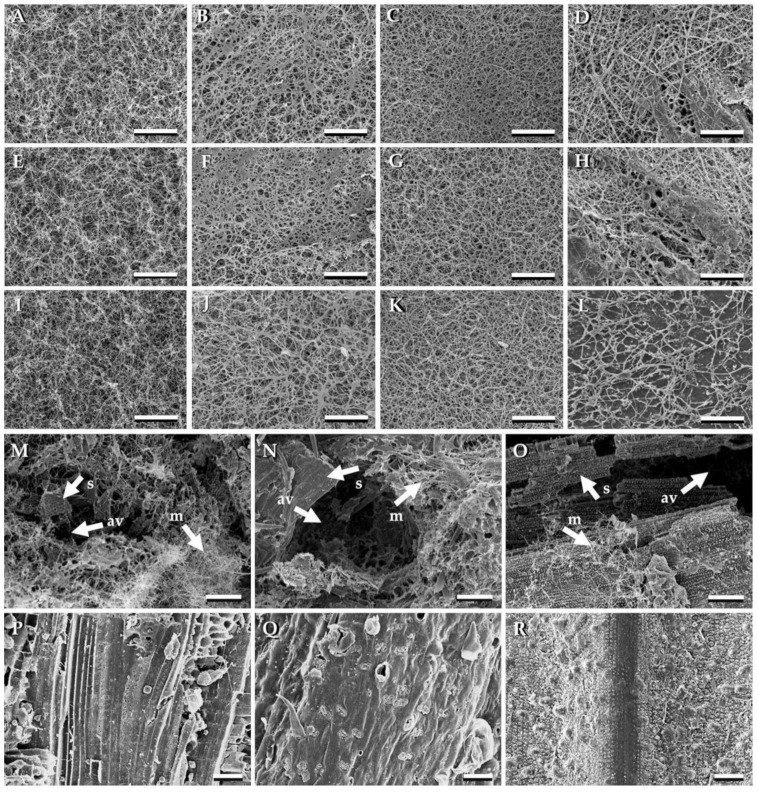
Scanning electron microscopic images of MBCs obtained in this study: The surfaces of MBCs produced from *Ganoderma fornicatum* with sawdust (**A**), corn husk (**E**), and rice straw (**I**). The surface MBCs produced from *Ganoderma williamsianum* with sawdust (**B**), corn husk (**F**), and rice straw (**J**). The surface MBCs produced from *Lentinus sajor-caju* with sawdust (**C**), corn husk (**G**), and rice straw (**K**). The surface MBCs produced from *Schizophyllum commune* with sawdust (**D**), corn husk (**H**), and rice straw (**L**). The cross sections of MBCs produced from *Lentinus sajor-caju* with sawdust (**M**), corn husk (**N**), and rice straw (**O**). The uncolonized sawdust (**P**), corn husk (**Q**), and rice straw (**R**). Arrows indicated substrate (s), fungal mycelia (m), and air-voids (av). Scale bar; (**A**–**O**) = 100 μm and (**P**–**R**) = 50 μm.

The morphological characteristics of MBCs were examined by SEM. It was found that the surfaces of all obtained MBCs were covered with fungal mycelia (Figure 2A–L). Based on a visual assessment of photographs in this study, MBCs made from *L. sajor-caju* and grown on all substrate types had a high degree of density in terms of mycelia when compared with other fungal species. Based on an assessment of the cross sections of MBCs, the fungal mycelia combined substrate particles through a series of mycelial networks and the air-voids present within the composites (Figure 2M–O). The morphological characteristics of the MBCs obtained in this study were similar to those described in several previous studies [26,42]. Remarkably, all uninoculated substrate types were observed to be absent of both fungal mycelium and air-voids (Figure 2P–R).

The moisture contents of the MBCs obtained in this study are shown in Table 1. The results indicate that these MBCs were comprised of a moisture content of 61.23 to 74.51% on a wet-mass basis. The moisture content varied depending on the fungal species and the type of substrate. The obtained moisture contents were within the ranges reported in previous studies at 59 to 80% on a wet-mass basis [43,44]. The results of this study agree with the findings of previous studies that reported that the moisture content of MBCs was dependent upon the fungal species and the type of substrate [43,44,45].

### 3.2. Determination of Physical Properties

#### 3.2.1. Density

The density values of the obtained MBCs in this study are shown in Table 1. The obtained density values ranged from 198.84 to 340.31 kg/m^3^. The highest degree of density of MBCs included in this study was found in MBCs produced from sawdust (318.59 to 340.31 kg/m^3^), followed by corn husk (220.74 to 240.99 kg/m^3^) and rice straw (198.84 to 222.76 kg/m^3^). MBCs made from *L. sajor-caju* and *G. williamsianum* exhibited higher degrees of density than the other fungal species. However, the lowest degree of density was obtained from MBCs made from rice straw and S. commune. It was found that the obtained density values in this study were within the ranges described in previous reports at 25 to 954 kg/m^3^ [19,21,26,35,36,39,46,47,48]. The results of this study are supported by the findings of several previous studies, which reported that the density of MBCs was significantly influenced by substrate type and fungal species [19,21,26,42]. This outcome is in accordance with Tacer-Caba et al. [47] who reported that the density of the MBCs produced from *A. bisporus*, *G. lucidum*, and *P. ostreatus* were grown on rapeseed cakes to produce MBCs with higher degrees of density than composites grown on oat husk. However, low-density values were observed in MBCs made from oat husk (25–38 kg/m^3^) and rapeseed cakes (41–58 kg/m^3^) derived from three different fungal species, all of which could potentially replace certain synthetic foams, namely, polystyrene (11–50 kg/m^3^), polyurethane (30–100 kg/m^3^), and phenolic formaldehyde resin foam (35–120 kg/m^3^) [19,20,21]. Moreover, several previous studies have reported that the pressing process (cold and/or heated pressing) significantly increased the resulting degree of density of MBCs [19,21,22,26,36,42,46]. Several previous studies [19,20,21] have also suggested that the high density (ranging from 440 to 680 kg/m^3^) of MBCs could potentially replace wood-based products (plywood, wood particle board, and wood insulation board). Moreover, the density of the MBCs was within the range of lignocellulosic materials (94–1560 kg/m^3^) [49,50]. In this study, the density of the obtained MBCs was similar to the density of polyimide foam (50 to 400 kg/m^3^) (Table 2).

#### 3.2.2. Water Absorption

**Figure 3:**
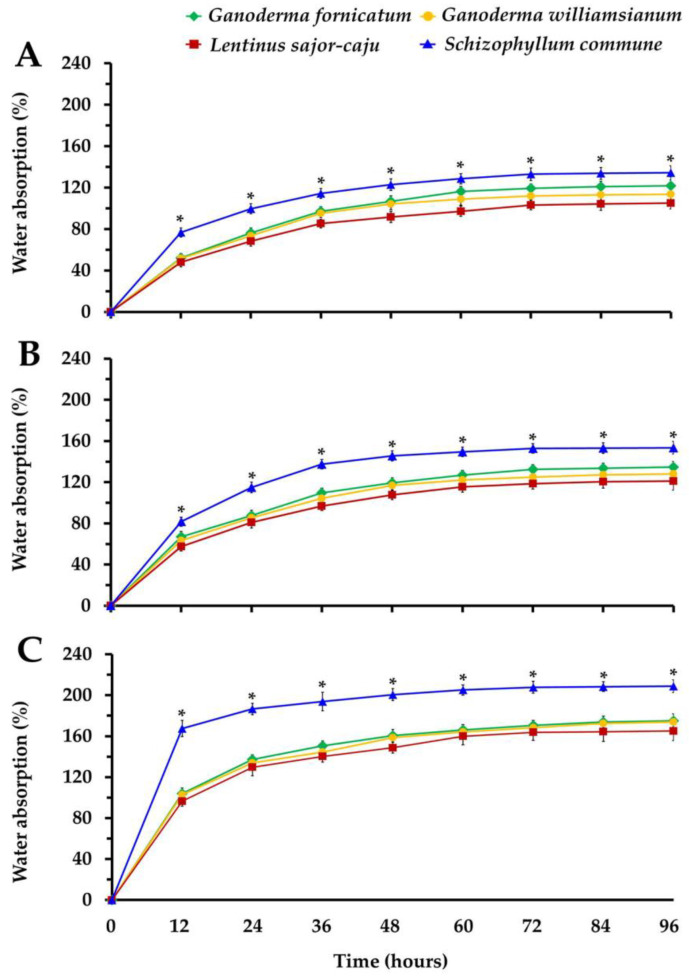
Water absorption ability of the obtained MBCs produced from sawdust (**A**), corn husk (**B**), and rice straw (**C**). Data are presented as means and the error bar at each point indicates the ± standard deviation. “*” indicates a significant difference according to Duncan’s multiple range test (*p* ≤ 0.05) at each point.

The water absorption ability of the obtained MBCs was determined by submerging the composites in water over a period of 96 h. It was found that the water absorption ability of the MBCs made from rice straw increased over a 24 h period and slowly stabilized after 36 h, whereas the water absorption ability of the MBCs made from corn husk and sawdust increased over a period of 60 h and slowly stabilized after 72 h (Figure 3). MBCs obtained from rice straw displayed the highest degree of water absorption ability, followed by MBCs obtained from corn husk and sawdust. The results also revealed that MBCs produced from *L. sajor-caju* exhibited lower water absorption ability in all the substrate types, while MBCs produced from *S. commune* exhibited a significantly high degree of water absorption ability in all substrate types. This study found that the water absorption ability decreased when the degree of density was increased. This result is in full agreement with the results of a number of previous studies, wherein the high degree of density of MBCs was reduced in accordance with their relevant water absorption ability [19,20,39,47]. After 96 h, the water absorption ability (105.07–208.82%) was observed to be within the ranges described in previous reports at 24.45 to 560% when left in contact with water for a period of 24–192 h (Table 2).

Several research studies have concluded that MBCs can be defined as hydroscopic materials, while the water absorption ability of MBCs was influenced by the type of substrate and fungal species. This characteristic is usually associated with a cellulose component (a large number of accessible hydroxyl groups) and hydrophilic mycelium [19,21,28,29,32,36,42,95,96,97]. Therefore, the differences in the water absorption abilities of various MBCs were found to be related to differences in the chemical components of the composites. Accordingly, the water absorption ability of MBCs in this study was influenced by the cellulose content in the substrate, of which rice straw was found to contain higher cellulose content (39–43% dry mass basis) than corn husk (30–35% dry mass basis) and sawdust (33–38% dry mass basis) [98,99,100,101]. In addition, Robertson et al. [102] found that the absorption ability of MBCs was reduced when smaller particle-sized substrates were used. An increase in the hydrophobic mycelia of *T. versicolor* on the surface of MBCs resulted in a lower degree of water absorption ability [39]. A comparison of the water absorption ability of the obtained MBCs and those of synthetic foams and wood-based composites is shown in Table 2. The water absorption ability of the obtained MBCs was within the ranges for wood particle and insulation boards, but was higher than that of synthetic foams and plywood products. Furthermore, the obtained MBCs also exhibited a water absorption capacity that was similar to those of lignocellulosic composites (53.6–148.8%) [103]. Remarkably, the high-water absorption ability of MBCs remains a major challenge in terms of the effective applications of these materials.

#### 3.2.3. Shrinkage

The low average shrinkage value of a material can contribute to the shape stability of the finished product [104]. In this study, MBCs obtained from rice straw exhibited the highest degree of shrinkage, followed by MBCs obtained from corn husk and sawdust (Table 1) when all fungal species were compared. MBCs obtained from *S. commune* in all three substrate types showed the highest degree of shrinkage. The lowest shrinkage value was observed in MBCs obtained from *L. sajor-caju* in all three substrate types, but these values were not determined to be significantly different from MBCs made from *G. fornicatum* and *G. williamsianum*. These results were supported by the outcomes of previous studies, which found that the shrinkage value of an MBC can vary depending on the substrate used [19,36,104]. The shrinkage values recorded in this study were within the range of some previously studied MBCs and those of wood insulation board (Table 2). Consequently, they could effectively be used to replace wood insulation boards.

#### 3.2.4. Thermal Degradation

**Figure 4:**
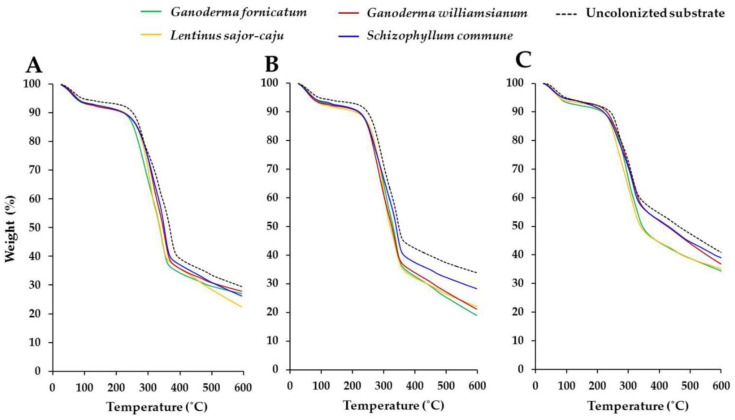
Thermogravimetric analysis of obtained MBCs produced from sawdust (**A**), corn husk (**B**), and rice straw (**C**).

All of the MBCs obtained in this study were found to be similar in terms of the degradation behavior of each lignocellulosic residue throughout all three stages of development (Figure 4). The first stage (free and chemically linked water evaporation resulting in about 5% mass loss) was observed to occur between 25 and 150 °C, followed by the second stage (degradation resulting in about 70% mass loss) and the third stage (decomposition), both of which were observed at temperatures in ranges of 200 and 325 °C and 350 and 375 °C, respectively. The results of this study were similar to the outcomes of several prior studies, which reported that the thermal degradation behavior of MBCs was comprised of three stages identified as the first, second, and third stages, and were recorded at temperatures within ranges of 25 to 200 °C, 200 to 375 °C, and at temperatures greater than 350 °C, respectively, all of which were related to the degree of thermal degradation of each used lignocellulosic residue [26,42,48,95,105,106,107,108]. This study found that each uncolonized lignocellulosic residue had a slightly slower mass loss than the MBCs in the same lignocellulosic residues. This outcome was in accordance with the findings reported by Appels et al. [26] and could be explained by the fact that fungal colonization can change the composition of lignocellulosic residues that are susceptible to thermal degradation.

This study found that the thermal degradation values of corn husk (containing 30–35% cellulose, 31–37% hemicellulose, and 8–14% lignin of dry mass basis) and sawdust (containing 33–38% cellulose, 29–31% hemicellulose, and 28–29% lignin of dry mass basis and 33–38% dry mass basis) were similar [98,99,100,101], but they differed from rice straw (containing 39–43% cellulose, 23–25% hemicellulose, and 12–20% lignin of dry mass basis). These results are supported by the findings of other previous studies, which reported that the type of lignocellulosic residues had no influence on thermal degradation due to the similar cellulose content [97,109,110,111]. The thermal degradation of MBCs was within the range of most synthetic foams and wood products; however, this was not the case for the thermal degradation of polypropylene and polyimide foams (Table 2).

### 3.3. Determination of Mechanical Properties

#### 3.3.1. Compression Strength

The results of this study found that the compression strength of the obtained MBCs varied depending on the different fungal species and the type of lignocellulosic residue (Table 3). MBCs produced from sawdust for each fungal species showed higher degrees of compression strength (1.59 to 1.87 MPa) than MBCs produced from corn husk (0.58 to 0.62) and rice straw (0.25 to 0.36 MPa). MBCs produced from *G. williamsianum* and *L. sajor-caju* exhibited higher degrees of compression strength when observed in the same substrate, while MBCs made from *S. commune* displayed the lowest degree of compression strength. The results of this study were in accordance with the outcomes of several prior studies, which found that the compression strength of MBCs varied based on the different fungal species and the type of lignocellulosic residue [19,21,22,36]. Tacer-Caba et al. [47] found that the compressive strength of MBC produced from *G. lucidum* grown on both oat husk and rapeseed cakes was higher than that of *A. bisporus* and *P. ostreatus* when grown on the same substrate. MBC produced by *P. sanguineus* grown on pine sawdust had a higher compression strength than *P. albidus* [48]. Angelova et al. [112] found that the compressive strength of MBC of *G. resinaceum* grown on rose flower residues (1.03 MPa) was significantly higher than when lavender straw (0.72 MPa) was used. Ghazvinian et al. [113] found that the MBC of *P. ostreatus* grown on sawdust (1.02 MPa) had a higher compressive strength than when straw was used (0.07 MPa). Additionally, Chan et al. [46] and Alemu et al. [18] found that the act of pressing during the production process effectively increased the compressive strength of MBCs.

The compression strength value of the obtained MBCs with synthetic foams and wood products is shown in Table 2. The obtained compressive strength in this study (0.25 to 1.87 MPa) was within the range of compressive strength obtained from previous reports (0.03 to 4.44 MPa). Moreover, the compression strength values of the obtained MBCs were within the range of synthetic foams, except for polypropylene, but were lower than the compression strength values of plywood and particle board products. Thus, the obtained MBCs might be appropriate for use in packaging and insulating applications that are typically associated with synthetic foams and wood fiber insulation boards.

#### 3.3.2. Tensile Strength

The tensile strength of MBCs obtained in this study was within the range of 0.20 to 0.87 MPa (Table 3). It was found that tensile strength was affected by the substrate type and the fungal species. MBCs produced from corn husk showed higher tensile strength than MBCs produced from rice straw and sawdust for all fungal species. Moreover, it was found that MBCs produced from *G. williamsianum* and *L. sajor-caju* displayed a relatively high degree of tensile strength. However, MBC made from *S. commune* had the lowest tensile strength among all substrates. These results were supported by the outcomes of several previous studies that found that the tensile strength of MBCs could be influenced by both the substrate type and the structure of the mycelium binder network [21,22]. Appels et al. [26] found that the tensile strength values of MBCs made from cotton in *P. ostreatus* were higher than those of rapeseed straw and beech sawdust. Accordingly, previous studies have reported that the tensile strength of MBCs can be influenced by the structure of the mycelium binder network, which varied depending on the type of mycelium network involved. Generally, monomitic species were associated with lower tensile strength values than dimitic and trimitic hyphal species [19,21,23,114]. Based on the three main hyphal types, only the monomitic species displayed generative hyphae, while the dimitic species formed two hyphal types (generative and skeletal hyphae), and the trimitic species were composed of all three types of hyphae. Accordingly, binding hyphae are thick-walled, dense, and hard, all of which contribute to the stiffness of the composite material [19,21,115,116]. Typically, *L. sajor-caju*, *G. fornicatum*, and *G. williamsianum* have a trimitic hyphal system, while *S. commune* has a monomitic hyphal system [117,118,119]. Therefore, MBCs made from *S. commune* in this study were associated with a low degree of tensile strength. Appels et al. [26] found that MBC of *T. multicolor* (trimitic hyphal system) exhibited higher tensile strength (0.04 MPa) than *P. ostreatus* (monomitic hyphal system) (0.01 MPa) when grown on rapeseed straw. Moreover, Appels et al. [26] found that the heat-pressing process resulted in the highest degree of tensile strength for MBCs, followed by the cold-pressing and non-pressing methods. The obtained tensile strength values of MBCs in this study were within the tensile strength values obtained from previous reports (0.01 to 1.55 MPa). These values were similar to those of polystyrene foam (0.15–0.7 MPa), phenolic formaldehyde resin foam (0.19–0.46 MPa), and polyimide foam (0.44–0.96 MPa) (Table 2). Therefore, the MBCs in this study may be used in the future to replace some synthetic foams and wood fiber insulation boards in packaging and insulation applications.

#### 3.3.3. Flexural Strength

The flexural strength values of MBCs obtained in this study are shown in Table 3. It was found that the highest degree of flexural strength of MBCs was found in the MBC produced from corn husk (0.18–0.32 MPa), followed by MBCs produced from rice straw (0.07–0.15 MPa) and sawdust (0.06–0.11 MPa), respectively. These obtained flexural strength values were within the ranges described in previous reports from 0.05 to 4.40 MPa (Table 2). MBCs produced from *G. williamsianum* and *L. sajor-caju* exhibited a high degree of flexural strength on all of the substrates used. The highest flexural strength of the obtained MBCs in this study was discovered in the MBC made from corn husk and *L. sajor-caju*. Appels et al. [26] and Jones et al. [21] suggested that the flexural strength of the MBC was dependent upon the type of mycelia network and substrate type, as well as the pressing method used. Lui et al. [120] and Chan et al. [46] found that the MBC made from *G. lucidum* grown on cotton stalks resulted in a higher flexural strength value than that of Chinese albizia sawdust. Moreover, MBCs made from *P. ostreatus* and rapeseed straw produced higher flexural strength values than when cotton was used [26].

Subsequently, the type of mycelium binder network used resulted in differing values of flexural strength [19,23,26]. Generally, MBCs produced from the trimitic fungal species exhibited higher flexural strength than the monomitic species, which was in accordance with their tensile strength behavior [20,22,114] and confirmed by the results of this study. *Trametes multicolor* (trimitic species) exhibited higher degrees of flexural strength than *P. ostreatus* (monomitic species) when rapeseed straw was used as a growing substrate [26]. Accordingly, the MBC exhibited a degree of flexural strength that was similar to that of polystyrene foam (0.07–0.70 MPa), but this flexural strength value was lower than polyimide foam, polyurethane foam, phenolic formaldehyde resin foam, polypropylene foam, and wood products (Table 2). Therefore, in terms of flexural strength, MBCs might not be appropriate for the structural applications that are generally associated with wood, but they may serve to replace some of the packaging materials that are currently made from polystyrene foam.

#### 3.3.4. Impact Strength

The impact strength values of MBCs in this study ranged from 0.21 to 2.70 kJ/m^2^ (Table 3). MBCs produced from corn husk showed higher impact strength than MBCs produced from rice straw and sawdust for all fungal species. MBCs produced from *G. williamsianum* and *L. sajor-caju* displayed higher degrees of impact strength than *G. fornicatum* and *S. commune* when all other substrates were used. However, there have been no previously published reports on the impact strength of MBCs. This study found that both the substrate type and the type of mycelia network had a considerable influence on impact strength. MBCs produced from trimitic fungal species were associated with a higher degree of impact strength than the monomitic fungal species due to the presence of thick-walled, dense, and hard hyphae. Moreover, previous studies reported that the differences in impact strength of the differing forms of lignocellulosic residues were dependent upon a number of other factors, such as fiber and matrix strength, load transfer efficiency, fracture propagation resistance, bonding strength, fiber distribution, and geometry [121,122]. In terms of impact strength, MBCs produced in this investigation were similar to polyimide foam, polyurethane foam, and phenolic formaldehyde resin foam (Table 2). Thus, the MBCs created in this study may be used to replace the synthetic foams that are used as insulation and packaging materials in the future.

### 3.4. Determination of Chemical Properties

**Figure 5:**
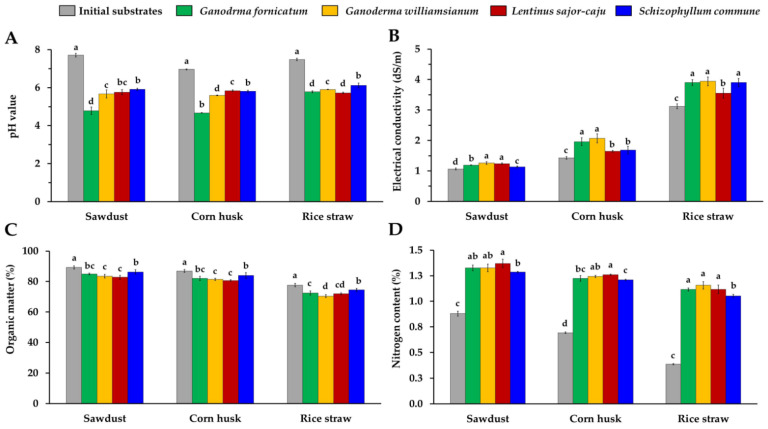
The pH value (**A**), electrical conductivity (**B**), organic matter content (**C**), and nitrogen content (**D**) of obtained MBCs and the initial substrates in this study. Data are presented as means and the error bar at each point indicates the ± standard deviation. Different letters in the same experiment of each substrate type are considered significantly different according to Duncan’s multiple range test (*p* ≤ 0.05).

In this study, the chemical properties, including pH value, electrical conductivity (EC), organic matter content, and nitrogen content of MBCs were investigated, and are presented in Figure 5. The initial pH values of the three growing substrates used, including sawdust, corn husk, and rice straw, were averaged at 7.71, 6.96, and 7.49, respectively. The final pH values of the obtained MBCs in the sawdust, corn husk, and rice straw were within ranges of 4.78–5.91, 4.67–5.81, and 5.28–6.12, respectively (Figure 5A). This indicates that the final pH values of MBCs were significantly decreased from the initial pH value of the growing substrate. This finding was in accordance with the outcomes of several prior studies, which reported that the finished MBCs exhibited lower pH values than the initial pH values of the growing substrates [18,39,41]. Attias et al. [41] found that the pH levels of MBCs produced from *P. ostreatus* and *P. salmoneo* (grown on woodchips of eucalyptus, pine, and apple trees) significantly decreased (4.3–4.7) when compared with the initial pH values of all selected substrates at around 5–5.5. This decrease in the pH values was generally caused by the enzymatic digestion process that took place during the growth of the fungal mycelia on the substrate [19,41].

The initial EC values in the sawdust, corn husk, and rice straw before inoculation were averaged at 1.08, 1.43, and 3.12 dS/m, respectively. After complete mycelium colonization, the EC values were significantly increased in all three of the substrates used (1.14–1.26 dS/m in sawdust, 1.65–2.07 dS/m in corn husk, and 3.55–3.94 dS/m in rice straw) (Figure 5B). Accordingly, Hwang et al. [123] reported that the EC value of the mycelium-colonized substrate (1.89 dS/m) was higher than that of the non-colonized substrate (1.12 dS/m). According to a number of previously published reports, an increase in the EC value in the substrate that had been colonized by mycelium could be explained by the fact that the substrate was degraded by an enzyme produced by the fungal mycelium, which also increased the amounts of inorganic compounds and minerals that were present, indicating a positive correlation with the EC value [98,124,125].

The percentages of the organic matter content of both the uncolonized substrate and MBCs are shown in Figure 5C. The results indicate that the organic matter content of the mycelium-colonized substrate was lower than the initial growing substrate. MBCs produced from *L. sajor-caju* and *G. williamsianum* exhibited low organic matter content in all of the substrates used. These results are supported by the findings of a number of previous studies, which found that the loss of organic matter in a substrate was caused by the enzymatic degradation of organic matter and the losses of CO_2_ and H_2_O that occurred during mycelial metabolism and development [39,41,126]. Moreover, Attias et al. [41] found that the mycelium colonization and development on a substrate are associated with the reduced amounts of organic matter in that substrate. Accordingly, a high degree of mycelium colonization and development resulted in low amounts of organic matter in that substrate.

The nitrogen content values of the initial growing substrate and the obtained MBCs are presented in Figure 5D. The results indicate that the nitrogen content of the obtained MBCs was significantly higher than that of the initial growing substrate among all types. The nitrogen contents of the obtained MBCs in this study (1.05 to 1.37%) were within the range of 0.5 to 1.6% as reported in previous studies (Table 2). Similarly, Attias et al. [39,41] found that the nitrogen content in MBCs was higher than for the control (non-colonized substrate) by a 1.0–1.7-fold increase. The change in nitrogen content that occurred during the mycelium growth process resulted from mycelium enzymatic digestion. Accordingly, nitrogen content is another factor that can be used to assess mycelium growth and development potential [18,19,21,41].

## 4. Conclusions

MBC production offers the advantage of using lignocellulosic residues to manufacture high-value-added products. In this study, three lignocellulosic residues and four different fungal species, along with their mechanical, physical, and chemical properties, were investigated for the purpose of developing MBCs. It was found that the properties of MBCs were directly affected by the type of substrate and fungal species involved. In terms of fungal species, MBCs of *G*. *williamsianum* and *L*. *sajor*-*caju* exhibited higher physical (high density, low water absorption, and low shrinkage) and mechanical properties (high compression, tensile, flexural, and impact strengths) than that of *G*. *fornicatum* and *S*. *commune*. In terms of substrate type, MBCs obtained from sawdust were associated with the highest degree of density and compression strength, and the lowest values in terms of both water absorption and shrinkage. However, MBCs made from corn husk exhibited the highest degrees of flexural, impact, and tensile strength. Additionally, the thermal degradation ability of MBCs was observed to be within a range of 200–325 °C, which was similar to the thermal degradation ability of lignocellulosic residues. Changes in the chemical properties of MBCs were typically caused by the enzymatic digestion processes associated with mycelial growth and the development of the fungus itself. The obtained MBCs were similar to those of polyimide and polystyrene foams based mainly on physical and mechanical data. However, problems of high water absorption and low impact strength were observed when compared with several synthetic foams. Low mechanical properties were observed when these composites were compared with wood products. Large-scale production remains a major challenge that will need to be addressed and improved in the future. Importantly, a deeper understanding of the biodegradable properties and the period of time required for the complete degradation of the MBC would result from further investigations. Additionally, further refinement of the standard analytical methods employed should take place in order to better evaluate the suitability of MBCs developed for each specific application, particularly for those used to produce packaging and insulation materials.

## Figures and Tables

**Table 1:** Moisture content, density, and average shrinkage of MBCs obtained in this study.

Substrates	Fungal Species		Parameters *	
		Moisture Content (%)	Density (kg/m^3^)	Shrinkage (%)
Sawdust	*Ganoderma fornicatum*	62.01 ± 0.64 b	337.21 ± 13.36 ab	8.86 ± 1.17 b
	*Ganoderma williamsianum*	61.34 ± 0.73 b	331.44 ± 9.39 a	8.40 ± 1.28 b
	*Lentinus sajor-caju*	61.23 ± 0.53 b	340.31 ± 16.41 a	8.10 ± 1.89 b
	*Schizophyllum commune*	64.23 ± 0.55 a	318.59 ± 8.14 b	10.83 ± 1.39 a
Corn husk	*Ganoderma fornicatum*	66.98 ± 0.51 b	232.11 ± 11.52 ab	12.64 ± 2.70 b
	*Ganoderma williamsianum*	65.11 ± 0.59 c	239.54 ± 8.65 a	12.32 ± 1.36 b
	*Lentinus sajor-caju*	64.94 ± 0.62 c	240.99 ± 15.61 a	11.91 ± 1.92 b
	*Schizophyllum commune*	70.22 ± 0.32 a	220.74 ± 11.22 b	15.27 ± 1.45 a
Rice straw	*Ganoderma fornicatum*	70.13 ± 0.65 bc	219.46 ± 8.29 a	14.26 ± 2.26 b
	*Ganoderma williamsianum*	69.55 ± 0.48 c	221.05 ± 15.01 a	13.95 ± 0.80 b
	*Lentinus sajor-caju*	70.48 ± 0.56 b	222.76 ± 2.81 a	13.26 ± 1.03 b
	*Schizophyllum commune*	74.51 ± 0.73 a	198.84 ± 10.17 b	16.31 ± 1.00 a

* The results are mean ± standard deviation. Different letters in the same column in each substrate type are considered significantly different according to Duncan’s multiple range test (*p* ≤ 0.05).

**Table 2:** Comparison of the properties of MBCs in this study with MBCs from previous studies, as well as synthetic foams and wood-based composites (modified from Aiduang et al. [19] and Jones et al. [21]).

Properties	MBCs		Products *							
			Synthetic Foams					Wood-Based Composites		
	This Study	Previous Studies	PI	PS	PU	PFR	PP	PW	PB	IB
D (kg/m^3^)	198.84–340.31	25–954	50–400	11–50	30–100	35–120	895–920	400–800	600–800	170–430
AS (%)	8.1–16.31	6.2–15.0	0.2–1.2	0.2–0.6	-	-	1.0–2.5	1–25	0.3–10	18.18–30.28
WP (%)	105.07–208.82	24.45–560	1.0–3.0	0.03–9	0.01–72	1–15	0.01–0.03	5–49	30.1–200	55–380
TD (°C)	200–325	225–375	474.1–546.8	318–440	278–379	270–475	360–460	250–380	310–350	150–270
CS (MPa)	0.25–1.87	0.03–4.44	0.6–1.4	0.03–0.69	0.002–48	0.2–0.55	31.19–48.29	8–25	1.8–3.4	0.1–1.21
TS (MPa)	0.20–0.87	0.01–1.55	0.44–0.96	0.15–0.7	0.08–103	0.19–0.46	31–41.4	10–44	10–100	0.35–1.38
FS (MPa)	0.06–0.32	0.05–4.40	0.59–1.36	0.07–0.70	0.21–57	0.38–0.78	22–23.2	35–78	1.5–7	2–2.5
IS (kJ/m^2^)	0.21–2.70	-	0.06–0.12	0.01–0.15	1.0–1.2	0.26–1.63	0.02–1	-	-	-

D = Density, AS = Average shrinkage, WP = Water absorption, TD = Thermal degradation, CS = Compression strength, TS = Tensile strength, FS = Flexural strength, IS = Impact strength, MBCs = Mycelium-based composites, PI = Polyimide, PS = polystyrene, PU = polyurethane, PFR = phenolic formaldehyde resin foam, PP = polypropylene, PW = plywood, PB = particle board, IB = insulation board and “–” = not reported. * Bruscato et al. [48], Yang [51], Omnexus [52], Du et al. [53], Shi et al. [54], Wang et al. [55], Wei et al. [56], Li et al. [57], Smirnov et al. [58], Dizon [59], Forest Products Laboratory [60], Stark et al. [61], Ashby [62], MatWeb LLC. [63], Azahari et al. [64], Filip et al. [65], NPCS Board of Consultants & Engineers [66], Niu and Wang [67], Jalalian et al. [68], Papadopoulou and Chrissafis [69], Tailor et al. [70], Deng et al. [71], Dou and Rodrigue [72], Zhu et al. [73], Shen et al. [74], Da Costa Castro et al. [75], Handayani et al. [76], Goulart et al. [77], Del Menezzi [78], Çolakoğlu and Colak [79], Jivkov et al. [80], Sinha et al. [81], Jamalirad et al. [82], Engineering Toolbox [83], Fateh [84], Zabihzadeh [85], STRUCTAflor [86], Mawardi et al. [87], Acda and Cabangon [88], Gößwald et al. [89], Ge et al. [90], Segovia et al. [91], Kallakas et al. [92], Harshavardhan and Muruganandam [93], and Muthuraj et al. [94].

**Table 3:** Compression, tensile, flexural, and impact strengths of MBCs obtained in this study.

Substrates	Fungal Species	Parameters *			
		Compression Strength (MPa)	Tensile Strength (MPa)	Flexural Strength (MPa)	Impact Strength (kJ/m^2^)
Sawdust	*Ganoderma fornicatum*	1.71 ± 0.03 b	0.34 ± 0.02 b	0.07 ± 0.00 bc	0.24 ± 0.00 b
	*Ganoderma williamsianum*	1.85 ± 0.01 a	0.42 ± 0.01 a	0.09 ± 0.02 ab	0.28 ± 0.02 a
	*Lentinus sajor-caju*	1.87 ± 0.03 a	0.44 ± 0.03 a	0.11 ± 0.02 a	0.30 ± 0.02 a
	*Schizophyllum commune*	1.59 ± 0.02 c	0.20 ± 0.01 c	0.06 ± 0.01 c	0.21 ± 0.02 b
Corn husk	*Ganoderma fornicatum*	0.59 ± 0.01 b	0.67 ± 0.04 bc	0.19 ± 0.01 b	2.05 ± 0.05 c
	*Ganoderma williamsianum*	0.62 ± 0.01 a	0.75 ± 0.06 b	0.28 ± 0.03 a	2.38 ± 0.12 b
	*Lentinus sajor-caju*	0.62 ± 0.02 a	0.87 ± 0.06 a	0.32 ± 0.02 a	2.70 ± 0.90 a
	*Schizophyllum commune*	0.58 ± 0.02 b	0.63 ± 0.06 c	0.18 ± 0.04 b	1.49 ± 0.08 d
Rice straw	*Ganoderma fornicatum*	0.33 ± 0.01 a	0.37 ± 0.04 b	0.10 ± 0.02 b	0.97 ± 0.10 a
	*Ganoderma williamsianum*	0.36 ± 0.02 a	0.46 ± 0.03 a	0.15 ± 0.03 a	0.99 ± 0.07 a
	*Lentinus sajor-caju*	0.33 ± 0.04 a	0.45 ± 0.02 a	0.16 ± 0.02 a	1.04 ± 0.08 a
	*Schizophyllum commune*	0.25 ± 0.03 b	0.35 ± 0.01 b	0.07 ± 0.01 b	0.68 ± 0.09 b

* The results are mean ± standard deviation. Different letters in the same column in each substrate type are considered significantly different according to Duncan’s multiple range test (*p* ≤ 0.05).

## Data Availability

Not applicable.
